# Changes in optic nerve sheath diameter and its correlation with degree of consciousness, pupil diameter, and light reflex in children with central nervous system infection after osmotherapy

**DOI:** 10.3389/fped.2024.1366968

**Published:** 2024-08-05

**Authors:** Anggia F. Rizqiamuti, Nelly A. Risan, Purboyo Solek, Dzulfikar D. L. Hakim, Harry G. Nugraha, Dewi Hawani, Mia M. Dewi, Elisabeth S. Herini

**Affiliations:** ^1^ Neurology Division, Department of Child Health, Faculty of Medicine, Universitas Padjadjaran/Hasan Sadikin General Hospital, Bandung, Indonesia; ^2^ Pediatric Emergency and Intensive Care Division, Department of Child Health, Faculty of Medicine, Universitas Padjadjaran/Hasan Sadikin General Hospital, Bandung, Indonesia; ^3^ Department of Radiology, Faculty of Medicine, Universitas Padjadjaran/Hasan Sadikin General Hospital, Bandung, Indonesia; ^4^ Neurology Divison, Department of Child Health, Faculty of Medicine, Gadjah Mada University/Sardjito Hospital, Yogyakarta, Indonesia

**Keywords:** CNS infection, increased ICP, osmotherapy, optic nerve sheath diameter, pediatrics

## Abstract

**Background:**

High intracranial pressure (ICP) is one of the most common complications of central nervous system (CNS) infection. Failure to control high intracranial pressure results in brain herniation and death. One of the treatments for high ICP involves the administration of osmotherapy in the form of 3% NaCl or 20% mannitol with observation during administration. Observation of ICP during administration of osmotherapy is possible through measurement of optic nerve sheath diameter (ONSD), which could be correlated with degree of consciousness, pupil diameter, and light reflex. Previous studies have not correlated ONSD with degree of consciousness, pupil diameter, and light reflex during the administration of osmotherapy.

**Purpose:**

To provide insights of incorporating ONSD measurement as a form of non-invasive bedside method for ICP monitoring by correlating it with degree of consciousness, pupil diameter, and light reflex at several time points.

**Methods:**

This study is a prospective cohort study, performed at Dr. Hasan Sadikin General Central Hospital Bandung, Cibabat General Regional Hospital, and General Regional Hospital Bandung Kiwari on children aged 2–18 years with decreased consciousness and CNS infection, from June 2023. Inter-rater reliability was performed with a correlation coefficient of 0.90. Measurement of ONSD, degree of consciousness, pupil diameter, and light reflex simultaneously up to 48 h after initiation of osmotherapy to 30 patients. Correlational analyses were performed using Spearman's rank.

**Results:**

Observation for 48 h after administration of osmotherapy showed changes in ONSD. A significant positive correlation was found between ONSD and degree of consciousness (r = 0.621 for the right eye and r = 0.602 for the left eye, *p* < 0.001). A significant positive correlation was found between ONSD and light reflex (r = 0.801 for the right eye and r = 0.812 for the left eye, *p* < 0.001). No significant correlation was found with changes of pupil diameter (r = −0.136 for the right eye and r = −0.141 for the left eye, *p* > 0.05).

**Conclusion:**

A significant correlation was found between ONSD and degree of consciousness and light reflex in children aged 2–18 years with CNS infection during administration of osmotherapy.

## 1 Introduction

Central nervous system (CNS) infection is a life-threatening disease and a potential threat toward public health, especially in children. The incidence of CNS infection in children is approximately 10.5–13.8/100,000, with case mortality of 30% and disability occurring in one-third of survivors. In 2010, the World Health Organization (WHO) reported that the types of CNS infection that often causes death in children are meningitis (422,900 cases) and encephalitis (143,500 cases) (1). A descriptive cross-sectional study in children with CNS infection at Hasan Sadikin General Central Hospital reported that 45.5% of the CNS infections are caused by tuberculosis (TB) infection, 21.3% caused by viral infection, 9.5% caused by bacterial infection, and 23.7% caused by non-specific infections (2).

One of the complications caused by CNS infection is increased intracranial pressure (ICP). Treatment of this condition should be administered as soon as possible because of the potential to cause herniation and death. Monitoring of ICP can be measured by invasive and non-invasive means. The gold standard for ICP monitoring consisted of invasive methods, such as the placement of a catheter in intraventricular, parenchymal, subarachnoid, and epidural spaces. There are several limitations of these methods, such as limited availability of equipment, the placement of catheters, which has to be performed by neurosurgeons, and complications such as infection and bleeding (3–5).

Alternatively, several non-invasive methods are available to assess degree of consciousness using the Glasgow Coma Scale (GCS), funduscopic examination, computed tomography (CT), magnetic resonance imaging (MRI), and measurement of optic nerve sheath diameter (ONSD) using ultrasonography (USG) (6–8). The limitation of ICP using degree of consciousness is relatively subjective and could cause difference in interpretation between examiners. The use of funduscopic examination for ICP assessment is inaccurate in acute situation, because it takes 7–10 days for papilledema to become clinically significant (9–11). The assessment of ICP using a head CT scan and MRI is constrained by limited resources and is difficult to perform on intubated patients (4, 7, 9, 12, 13). The measurement of ONSD is one of the non-invasive methods that can be performed as an alternative to invasive methods, because it can be performed bedside, with comparable results to invasive methods as well as a sensitivity and specificity of more than 90% (14, 15).

A study involving 30 children with head injuries in India showed that patients were considered to have ICP if the ONSD is more than 4 mm for children aged below 1 year, more than 4.5 mm for children aged above 1 year, and more than 5 mm for children aged above 15 years (14, 16). Serial prospective research in India involving 48 children aged 7.5–12 years reported that ONSD in patients with head injury and without head injury, most of which were due to CNS infection, reported that the ONSD cut-off value for increased ICP was 4 mm for children aged below 1 year, 4.71 mm for children aged 1–10 years, and 5.43 for children aged above 10 years. This research also reported that the assessment of ICP using ONSD has a sensitivity of 100% and specificity of 66.7%. Approximately 85% of patients with a GCS of 12 showed increased widening of ONSD (17, 18). A cross-sectional study at Hasan Sadikin General Central Hospital involving 32 children with increased ICP due to CNS infection reported that the widest ONSD was found in patients with tuberculous meningitis (TBM) grade III with a mean left and right ONSD of 6 mm (19).

Measurement of pupillary diameter and light reflex can be used for ICP monitoring. Increased ICP can cause changes in pupillary diameter (<2 or >5 mm) accompanied by slowed or lost pupillary response toward light (20). A prospective cohort study involving children aged less than 18 years with acute head injury and encephalopathy showed a negative correlation between increased ICP and measurement of pupillary diameter (21). Pupillary light reflex is inversely correlated with increased ICP and decreased pupil reactivity (22).

One of the treatments for increased ICP is the administration of osmotherapy, which creates an osmotic gradient resulting in the movement of fluid from the cerebrospinal fluid compartment into the blood vessel. The most commonly used forms of osmotherapy in our hospital are 20% mannitol and 3% NaCl (23). The administration of osmotherapy results in decreased ONSD, improvement of consciousness, normal pupil diameter (2–5 mm), and immediate return of light reflex. A randomized controlled trial involving 50 participants with head injury showed no difference in ONSD between the patient group who were given continuous 0.5 ml/kg/h of 3% NaCl and bolus of 3% NaCl 3 ml/kg/h for 48 h (24). An observational prospective study comparing the effectivity of 3% NaCl and 20% mannitol on head injury patients with increased ICP reported a significant decrease in ONSD upon observation at 6, 12, 24, and 48 h after administration of 3% NaCl and 20% mannitol (25).

Monitoring ICP during the administration of osmotherapy is important. Therefore, our study aimed to provide insights into incorporating ONSD measurement as a non-invasive bedside method of ICP monitoring by correlating it with degree of consciousness, pupil diameter, and light reflex at several time points.

## 2 Methods

### 2.1 Patient selection and study design

This was a prospective cohort study. The participants recruited for this study were patients aged 2–18 years with decreased consciousness and suspected of having CNS infection. These patients were recruited by consecutive sampling. Participants with disorders of the eye, such as glaucoma, retro-orbital infection, conjunctivitis, cataract, postorbital enucleation, and intraocular mass, were excluded. Patients with glucose >250 mg/dl, natrium serum >160 mg/dl, and space-occupying lesion were also excluded. Upon further examination and during the follow-up period, if patients had a CT scan result of obstructive hydrocephalus and underwent placement of a ventriculoperitoneal shunt (VP shunt), the patient was dropped out of the study. If a patient’s family decided to discharge the patient from hospital, the patient died, or the natrium serum value <125 or >160 mEq/L, they were also dropped out of the study.

(1) $$n=\frac{{\left(1.65+0.84\right)}^{2}}{{\left\{0.5\;\ln\left(\frac{1+0.476}{1-0.476}\right)\right\}}^{2}}+3$$

The sample size calculation for the correlational analysis was performed with α of 5% (Z*_α_* = 1.65; one-tailed test), test power of 80% (Z*_β_* = 0.84), and using a study by Kavak et al. (26) as a reference correlational coefficient (r = −0.476), as shown in Equation 1. As a precaution for dropouts, 10% of the calculated number was added, resulting in 30 as the final number of samples. The patients in this study were those referred to the emergency department of Hasan Sadikin General Central Hospital, Cibabat Regional Hospital, and Bandung Kiwari Regional Hospital, where the study was conducted.

### 2.2 Measurement of degree of consciousness, pupil diameter, light reflex, and optic nerve sheath diameter

Upon admission, the patient underwent history taking and physical examination, consisting of degree of consciousness, vital signs, complete neurological examination, and anthropometric status. The measurement of ONSD and serum natrium level were performed before the administration of osmotherapy. Other supporting examinations, such as urea, creatinine, serum glutamic oxaloacetic transaminase (SGOT)/serum glutamic pyruvic transaminase (SGPT), random blood sugar, chest radiography, and Mantoux test, were performed on indication. If the serum natrium level was ≤125 mEq/L, the patient received correction of serum natrium until the level increased above 125 mEq/L and measurements for the study were commenced.

Patients with a serum natrium level above 135 mEq/L will be given 20% mannitol with a starting dose of 0.5 g/kg, increased incrementally up to 1 g/kg and repeated every 6 h. Measurement of ONSD, degree of consciousness, pupil diameter, and pupil light reflex were performed before osmotherapy with 20% mannitol and at 1, 12, 24, and 48 h after osmotherapy. Patients with a serum natrium level in the range of 125–134 mEq/L will be given NaCl 3%. Measurement of ONSD, degree of consciousness, pupil diameter, and pupil light reflex were performed before osmotherapy with 20% mannitol and at 10 min, 12, 24, and 48 h after osmotherapy. After 24 h of osmotherapy, the measurement of serum natrium level was repeated and reassessed. If the serum natrium rose above 135 mEq/L, osmotherapy will be switched from 20% mannitol to NaCl 3%. If the serum natrium dropped below 135 mEq/L, osmotherapy will be switched from 3% NaCl to mannitol 20%. If upon 24 h of observation after osmotherapy, improvements of degree of consciousness, pupil diameter, and light reflex were found, osmotherapy administration will be stopped.

**Figure 1:**
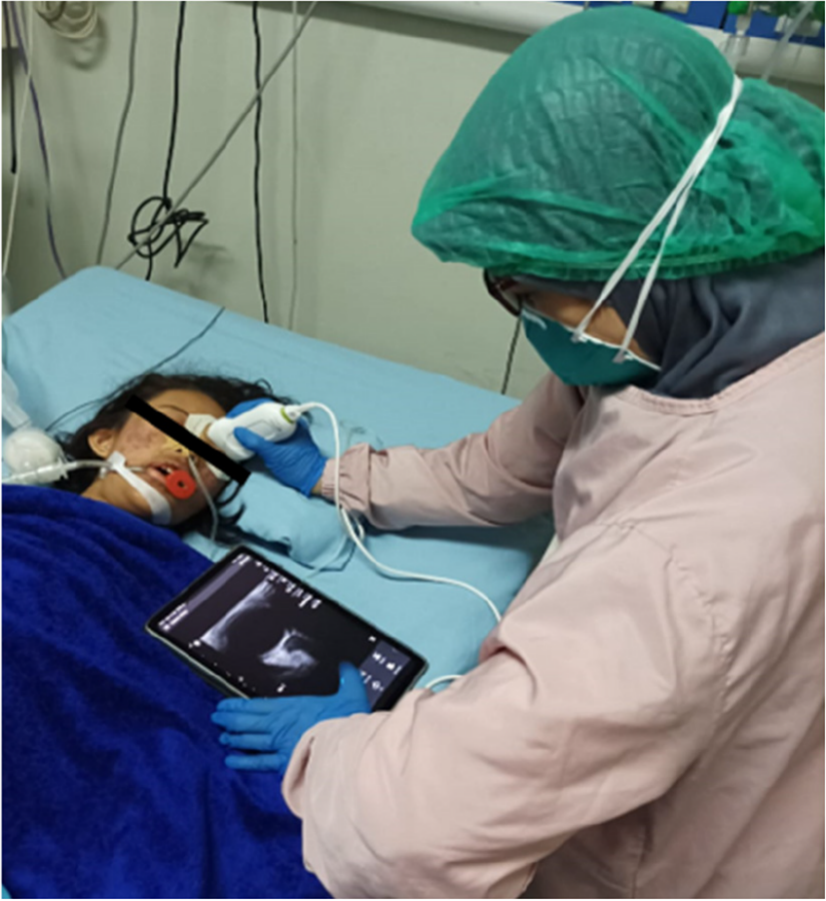
Patient position during measurement by a pediatrician.

**Figure 2:**
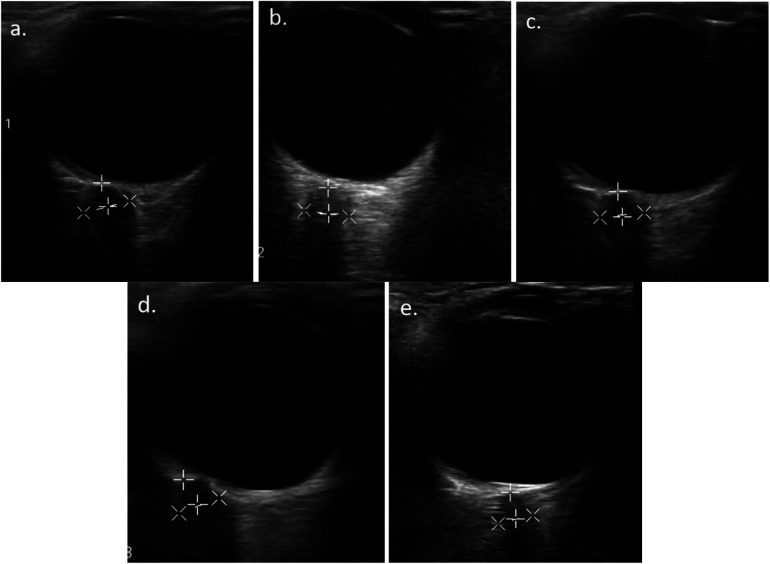
Serial ONSD measurement result of the right eye of one of the patients: (**A**) before mannitol administration (6.7 mm), (**B**) 1 h (5.33 mm), (**C**) 12 h (5.28 mm), (**D**) 24 h (4.78 mm), and (**E**) 48 h post mannitol administration (4.21 mm).

The measurement of ONSD was performed by a pediatrician using an Android/iOS-based Philips Lumify Ultrasound System (USA) linear type (L12-4), 7.5/10 MHz at the patient's bedside (Figure 1). The patients were positioned in a supine position with their head held up at an angle of 30°. The patient's left and right eyes were closed alternatingly with transparent tape. The ultrasound probe with applied gel was placed above the patient's eyelids without pressure. The measurement of ONSD was performed alternatingly for each eye and repeated three times for each eye using the horizontal view. However, in the case of poor visualization with the horizontal view, the sagittal view was used. The optic nerve was visible as a linear hypoechoic structure at the posterior margin of eye socket (Figure 2).

The measurement of pupil diameter was performed in a room with adequate lighting. The patient's eye was opened and light was directed toward the pupil from underneath the patient's nose. The diameter of each pupil was measured using a ruler. A difference in pupil diameter below 1 mm was considered normal.

The measurement of light reflex included both direct and consensual light reflex. A light reflex examination was performed using a pen light as a stimulus for 2–3 s while observing the constriction reflex of the right eye and observing the direct and consensual reflex of the right eye. The pen light was moved swiftly toward the left eye past the nose. The direct and consensual reflexes of the left eye were immediately examined.

### 2.3 Statistical analysis

The obtained numeric data were assessed for normality of distribution using the Shapiro–Wilk test. Data with a *p*-value >0.05 were considered normally distributed. The analysis of ONSD changes was performed with repeated measures ANOVA if the data were normally distributed and the Friedman test if the data were not normally distributed. An analysis of correlation between ONSD changes, degree of consciousness, and pupil diameter was performed using the Rank Spearman correlation. All analysis of the data was performed using SPSS version 25 (IBM Corp., New York, USA) with a significance value of *p* < 0.05.

Apart from the data analysis, the interrater reliability of the examiner for ONSD measurement was also assessed using Guildford Correlation. One of the examiners was a pediatric radiology consultant as the expert and the other was a pediatrician. The measurement of ONSD for this assessment was performed for 30 children aged 2–18 years, with or without CNS infection. The result of the assessment showed the measurements performed by both examiners were similar (r = 0.990 for the right eye, r = 0.999 for the left eye, *p* > 0.05).

## 3 Results

The patients included in this study, between June and October 2023, consisted of 13 patients from Hasan Sadikin General Central Hospital, 16 patients from Cibabat Regional Hospital, and 1 patient from Bandung Kiwari Regional Hospital. The characteristics of patients included in this study are displayed in Table 1.

**Table 1:** Characteristics of patients (*n* = 30).

Characteristics	Frequency
Sex	
Male	10 (33.3%)
Female	20 (66.7%)
Age (years)	
2–10	18 (60%)
11–18	12 (40%)
Mean (SD)	8.4 (4.1)
Range	2–15
BMI (kg/m^2^)	
Mean (SD)	14.70 (2.44)
Range	10.63–23.24
Nutritional status	
Severe thinness	3 (10.0%)
Moderate thinness	2 (40%)
Normal	14 (46.7%)
Obesity	1 (3.3%)
Degree of consciousness pre osmotherapy	
Coma	7 (23.3%)
Stupor	23 (76.7%)
ONSD pre osmotherapy	
Right: mean (SD)	5.46 (0.37)
Left: mean (SD)	5.51 (0.36)
ONSD pre osmotherapy according to age	
Right: mean (SD)	
Age 2 to <15 years old	5.44 (0.36)
Age 15 to 18 years old	5.63 (0.66)
Left: mean (SD)	
Age 2 to <15 years old	5.50 (0.34)
Age 15 to 18 years old	5.62 (0.73)
Pupil diameter (mm) pre osmotherapy	
Right: mean (SD)	3.12 (1.08)
Left: mean (SD)	3.22 (1.08)
Light reflex pre osmotherapy	
Right	
Slow	27 (90%)
Reactive	3 (10%)
Kiri	
Slow	27 (90%)
Reactive	3 (10%)
Received osmotherapy	
NaCl 3%	21 (70%)
Mannitol 20%	9 (30%)

The patients who received 3% NaCl and 20% mannitol showed no significant difference in characteristics, degree of consciousness, ONSD, and pupil diameter (*p* > 0.05). Therefore, further analyses were performed by combining the two groups.

The optic nerve sheath diameter of both eyes at each time point of measurement showed a significant difference, with a decreasing trend toward the last measurement (Table 2). The median ONSD before osmotherapy was 5.49 mm (range 4.46–6.14 mm) for the right eye, whereas the median ONSD for the left eye was 5.57 mm (range 4.79–6.38 mm). At 48 h after osmotherapy, the median ONSD for the right eye was 4.64 mm (range 3.38–6.58 mm), whereas the median for the left eye was 4.64 mm (range 3.60–6.56 mm).

**Table 2:** Changes of ONSD in each time point of measurement.

	ONSD of right eye		ONSD of left eye	
Measurement	Median (range)	*p*-value^a^	Median (range)	*p*-value^a^
I	5.49 (4.46–6.14)	<0.001	5.57 (4.79–6.38)	<0.001
II	5.22 (4.57–6.79)		5.26 (4.61–7.25)	
III	5.12 (4.23–6.37)		5.10 (4.54–6.80)	
IV	4.91 (3.90–6.27)		4.99 (3.78–6.42)	
V	4.64 (3.38–6.58)		4.62 (3.60–6.56)	
Comparison		*p*-value^b^		*p*-value^b^
I vs. II		0.004		<0.001
II vs. III		0.047		0.058
III vs. IV		0.048		0.017
IV vs. V		0.010		0.038

^a^ Friedman test.

^b^ Wilcoxon test.

The pupil diameter of both eyes showed no significant difference at each time point of measurement (Table 3). The median pupil diameter before osmotherapy was 3 mm (range 0.5–5 mm) for the right eye, whereas the median for the left eye was 3 mm (range 0.5–5 mm). At 48 h after osmotherapy, the median ONSD for the right eye was 3 mm (range 2–5 mm), whereas the median for the left eye was 3 mm (range 2–5 mm).

**Table 3:** Changes of pupil diameter in each time point of measurement.

	ONSD of right eye		ONSD of left eye	
Measurement	Median (range)	*p*-value^a^	Median (range)	*p*-value^a^
I	3 (0.5–5)	0.408	3 (0.5–5)	0.458
II	3 (0.5–6)		3 (0.5–5)	
III	3 (1–5.5)		3 (1–5)	
IV	3 (2–5)		3 (2–5)	
V	3 (2–5)		3 (2–5)	
Comparison		*p*-value^b^		*p*-value^b^
I vs. II		0.149		0.258
II vs. III		0.918		0.877
III vs. IV		0.233		0.609
IV vs. V		0.123		0.230

^a^ Friedman test

^b^ Wilcoxon test.

Degree of consciousness in patients at each time points of measurement showed a significant difference, with a trend of increasing frequency of patients with higher degrees of consciousness toward the last measurement (Table 4). Before osmotherapy, there were 7 coma patients and 23 stupor patients. After 48 h of osmotherapy, there were 8 coma patients, 7 stupor patients, 11 somnolent patients, and 4 compos mentis patients.

**Table 4:** Changes in degree of consciousness in each time point of measurement.

Degree of consciousness	Measurement						
	I	II	III	IV	V	Comparison	*p*-value^a^
Coma (GCS 3–8)	7	7	8	8	8	I vs. V	0.002
Stupor (GCS 9–12)	23	23	22	20	7	II vs. V	0.001
Somnolent (GCS 13–14)	—	—	—	2	11	III vs. V	0.001
Compos mentis (GCS 15)	—	—	—	—	4	IV vs. V	0.001

^a^ Wilcoxon test.

The light reflex in both eyes showed a significant difference at several time points, with an overall increase in the frequency of patients with returning light reflex (Table 5). Before osmotherapy, 26 patients had slow reflexes and 4 patients had non-reactive light reflexes. After 48 h of osmotherapy, 14 patients had an improved rate of light reflex.

**Table 5:** Changes in light reflex in each time point of measurement.

	Measurement						
Light reflex	I	II	III	IV	V	Comparison	*p*-value^a^
Right eye						I vs. IV	0.025
Fast (1)	—	—	—	3	14	I vs. V	**0**.**001**
Slow (2)	26	27	28	25	12	II vs. IV	0.102
Non-reactive (3)	4	3	2	2	4	II vs. V	**0**.**003**
						III vs. IV	0.180
						III vs. V	**0**.**007**
						IV vs. V	**0**.**020**
Mata kiri						I vs. IV	0.102
Fast (1)	—	—	—	3	14	I vs. V	**0**.**003**
Slow (2)	26	27	27	24	11	II vs. IV	0.257
Non-reactive (3)	4	3	3	3	5	II vs. V	0.007
						III vs. IV	0.180
						III vs. V	**0**.**007**
						IV vs. V	**0**.**020**

^a^ Wilcoxon test.

*p*-value <.05 means the results are statistically significant.

In our study, 26 of the patients received sedative agents, such as midazolam, phenobarbital, phenytoin, and diazepam, which might have had an effect on the measurement results. We split the patients into two groups (sedative agents vs. no sedative agents) and compared degree of consciousness, ONSD, pupil diameter, and light reflex of both eyes before and 48 h after osmotherapy. There was no significant difference between the two groups (*p* > 0.05).

The results of the correlation analysis between ONSD with changes in pupil diameter, degree of consciousness, and light reflex are displayed in Table 6. A significant positive correlation was found between ONSD changes and degree of consciousness during the course of osmotherapy at 48 h (r = 0.621 for the right eye, r = 0.602 for the left eye, *p* < 0.001). A significant positive correlation was also found between ONSD changes and light reflexes 48 h after osmotherapy (r = 0.801 for the right eye and r = 0.812 for the left eye, *p* < 0.001). No significant correlation was found between ONSD and changes of pupil diameter (r = −0.136 for the right eye and r = −0.141 for the left eye, *p* > 0.05).

**Table 6:** Correlation between ONSD and pupil diameter, degree of consciousness, and light reflex.

	Right eye		Left eye	
Correlation between ONSD and	r	*p*-value	r	*p*-value
- Changes of pupil diameter	−0.136	0.473	−0.141	0.294
- Degree of consciousness pre osmotherapy	0.305	0.101	0.276	0.137
- Degree of consciousness post osmotherapy	**0**.**621**	**<0**.**001**	**0**.**602**	**<0**.**001**
- Light reflex pre osmotherapy	0.193	0.308	0.198	0.294
- Light reflex post osmotherapy	**0**.**801**	**<0**.**001**	**0**.**812**	**<0**.**001**

r = coefficient of correlation calculated by Spearman's rank.

Decrease of ONSD: median (range): right eye: 0.78 (−1.29 to 0.22); left eye: 0.72 (−1.48 to 2.06); decrease of pupil diameter: median (range): right eye: 0.00 (−2.5 to 3.0); left eye: 0.00 (−2.5 to 3.0).

*p*-value <.05 means the results are statistically significant.

## 4 Discussion

The participants who fulfilled our inclusion and exclusion criteria were 30 children [mean age 8.4 years (SD 4.1)]. These children came from three different hospitals: 13 from Hasan Sadikin General Central Hospital, 1 from Bandung Kiwari Regional Hospital, and 16 from Cibabat Regional Hospital. The distribution of degree of consciousness in patients upon admission was stupor in 23 (76.7%) children and coma in 7 (23.3%) children. An observational prospective study in India involving 50 children aged 1 month to 12 years with CNS infection showed that 6% of the patients came with coma (27). A cross-sectional study with 45 children aged below 18 years with CNS infection reported that 17 (38%) children were compos mentis, 5 (11%) were somnolent, and 10 (22%) were in a coma (28). The results found in this study differed from those two studies because the age range for recruitment in this study was for children aged above 2 years and needed osmotherapy. Osmotherapy is generally administered for patients with a GCS score <8 (29). However, in this study, patients with a GCS score  ≤ 13 were given osmotherapy because the ONSD was >4.5 mm, the pupil diameter was <2 or >5 mm and had slowed pupillary reflex (16, 30). The participants who received osmotherapy in this study showed an improvement of ONSD, pupillary diameter, and light reflex.

A study from Turkey comparing ONSD in healthy boys and girls reported that ONSD increases with increasing age, up to the age of 15 years (31). Another study involving 102 children aged 0–15 years reported normal ONSD values according to age in children. The normal ONSD value is 2.1–4.0 mm for children aged under 1 year and 2–3 mm for children aged above 1 year (32). A cross-sectional study involving participants aged 2–14 years in Nigeria showed no correlation between ONSD, body mass index (BMI), and sex in healthy children (33). It is important to note that in that study, all the participants had increased ICP, which makes it difficult to assess whether there are correlations between BMI, age, and sex.

The optic nerve sheath is connected to the subarachnoid space, which means that ONSD will change according to changes in ICP (34). Other factors affecting changes in ONSD include the duration of increased ICP, the intraocular pressure before increased ICP, and blockage in subarachnoid spaces as well as the degree of brain tissue damage. The measurement of ICP has to be performed carefully in children aged below 1 year, because the anterior fontanelle has not yet closed and will affect the changes in ONSD. According to several prior studies, the upper normal limit of ONSD in children aged below 1 year is 4 mm, whereas children aged above 1 year have an upper normal limit of 4.5 mm (6, 35). A study that grouped together increased ONSD according to age showed that for children aged below 1 year, ONSD ≥5.16 mm is considered to be increased ICP at ≥20 mmHg, whereas for children aged above 1 year, ONSD ≥5.75 mm is considered to be increased ICP at ≥20 mmHg (36). In our study, the participants were aged 2–12 years with the consideration that anterior fontanelle had closed and changes in ONSD would correlate more with increased ICP.

A study involving 174 children with increased ICP due to head trauma showed that an ONSD value >5.5 mm was equivalent to an ICP of 20 mmHg using an invasive method of measurement (37). A study involving 48 patients in the intensive care unit (ICU) diagnosed with CNS infection reported that the ONSD value for increased ICP is 4.64 ± 0.48 mm for infants, 6.4 ± 0.65 mm for children aged 1–10 years, and 6.28 ± 0.62 mm for children aged above 10 years. It was concluded that the ONSD value for increased ICP in participants aged below 1 year is >4.0 mm, 4.71 mm for participants aged 1–10 years, and 5.43 mm for participants aged above 10 years with a sensitivity of 100% and specificity of 60%. Of the patients in this study, 85% showed increased ICP with ONSD with a GCS of 12 (stupor) (38). The results of our study are consistent with those of previous studies. The mean ONSD at the beginning of our study for patients aged 2–14 years was 5.44 mm (SD 0.36) for the right eye and 5.5 mm (SD 0.34) for the left eye; for patients aged 15–18 years, it was 5.63 mm (SD 0.66) for the right eye and 5.62 mm (SD 0.73) for the left eye.

A study from Hasan Sadikin General Central Hospital in 2022 of participants aged 2–18 years with CNS infection showed that the widest ONSD was found in TBM grade III patients, with the mean ONSD for the right and left eyes exceeding 6 mm (19). In that study, the measurement was only performed once and no osmotherapy was administered. The ONSD results in our study showed that participants with grade 3 TBM had a mean ONSD of 5.81 mm for the right eye and 5.83 mm for the left eye, before the administration of osmotherapy. After osmotherapy administration, the mean ONSD for the right and left eyes were 5.86 and 5.88 mm, respectively. A total of nine children had grade 3 TBM, three of whom were brain dead, while others showed an improvement in ONSD value, degree of consciousness, pupil diameter, and light reflex.

Osmotherapy, which was administered in this study, includes 3% NaCl and 20% mannitol with different administration times. However, no significant difference was found in terms of degree of consciousness, ONSD, pupil diameter, and light reflex in each measurement time point between the two osmotherapy groups (*p* > 0.05). Therefore, the study proceeded without further comparison between the two groups.

Studies of children are still limited, and there are no other prospective cohort studies that look into the changes in ONSD after osmotherapy administration by performing continuous measurements. To the best of our knowledge, ours is the first study on patients with CNS infection. In our study, ONSD was positively correlated with the degree of consciousness (r = 0.621 for the right eye, r = 0.602 for the left eye, *p* < 0.001). The median ONSD before osmotherapy was 5.49 mm (range 4.46–6.14 mm) for the right eye and 5.57 mm (range 4.79–6.38 mm) for the left eye. After 48 h of osmotherapy administration, the mean ONSD of the right and left eye was 4.64 mm (SD 3.38) with range of 3.60–6.56 mm. The results of our study are in accordance with those of several previous studies. A study involving children with brain parenchymal injury and closed head trauma without head CT scan abnormalities showed that ONSD was wider in children with consciousness in the category of stupor to coma (GCS 3–13) compared to somnolent and compos mentis (GCS 14–15) (13). Another study involving 68 participants aged 0–60 months experiencing head trauma with increased ICP and 226 healthy controls reported that the ONSD in participants with moderate and severe head injury was wider [5.3 mm (SD 1.1)] compared to mild head injury [4.1 mm (SD 1)] (13). A prospective study involving 162 adults with head injury reported that changes in ONSD were associated with degree of consciousness (*p* < 0.001) (39). A study involving 76 individuals aged above 18 years with head trauma and 65 participants without head trauma compared the degree of consciousness and ONSD, and reported a decreased ONSD and improved degree of consciousness [11 (SD 2.8) vs. 12.8 (SD 2.9), *p* < 0.0001] after 48 h of osmotherapy (40). An observational prospective study on 30 adults with head trauma reported an improvement after 48 h of osmotherapy using 20% mannitol. Before osmotherapy, the GCS was in the range of 4–12 and the ONSD was in the range of 5.6–7.2 mm. After administration of osmotherapy, the mean ONSD was 4.8 mm and the GCS was 13 (25). These findings meant that an early diagnosis of increased ICP can affect the prognosis of the patient. A study involving 40 participants with head injury and subarachnoid bleeding after 20% mannitol administration [dose 0.54 (0.49–0.80) g/kg] showed that all the participants experienced a drop in ONSD [from 6.3 (6.1–6.7) mm to 5 (5.5–6.3) mm, *p* = 0.007] (41).

Four participants in our study were in a coma for more than 48 h, reached the health facilities after more than 3 days, and died after reaching the health facilities. The mean ONSD before osmotherapy for the right eye was 5.73 mm (range 5.14–6.13 mm) and for the left eye was 5.82 mm (range 5.08–6.13 mm). After osmotherapy, the mean ONSD for the right eye was 6.38 mm (range 6.13–6.57 mm) and for the left eye was 6.42 mm (range 6.33–6.56 mm). During the course of the treatment, the patient was declared brain dead and died after 48 h of therapy. This finding suggested that increased ONSD in the early course of osmotherapy and the duration of brain injury determine the prognosis. The four participants were known to have headaches for 1 week before hospital admission, and the ONSD was still widened after osmotherapy. However, only one participant underwent a head CT scan and showed communicating hydrocephalus, whereas the other three did not undergo head CT scan due to their condition. This finding is consistent with a prospective cohort study at Sanglah General Hospital on children with CNS infection (encephalitis and meningitis) and head trauma. That study reported that the participants receiving 20% mannitol for more than 24 h after the onset of decreased consciousness had a poorer prognosis (42). An observational prospective study in India on adult patients with head injury reported that compared to the controls, head injury patients had a higher ONSD (4.8 vs. 3.4 mm, *p* = 0.001). It was also reported that the measurement of ONSD at 24–48 h after the onset of decreased consciousness had a better prognosis compared to those performed more than 48 h after the onset of decreased consciousness (43).

A study looking at ONSD changes in children with varying degrees of increased ICP reported that ONSD in the brain dead group was higher than in the group with only increased ICP and normal ICP [mean 5.33 mm (SD 0.62), 5.11 mm (SD 0.46), and 4.31 mm (SD 0.5), respectively] (44). A previous study of 21 brain dead adults reported that the mean ONSD after the individuals were declared brain dead was significantly higher than before they were declared brain dead [4.339 mm (SD 0.285) vs. 5.952 mm (SD 0.464)] (45). These results showed that ONSD will reach a maximum diameter and will have difficulties returning to normal upon the occurrence of brain death.

Pupil reactivity and diameter are important components to assess the function of the brainstem in relation to increased ICP (46). Increased ICP will affect the tract of cranial nerve II (optic nerve) and cranial nerve III (oculomotor nerve), which can be evaluated by measuring the pupil diameter and light reflex. Both these examinations can be performed using a pen light or quantitative automated pupillometry. A normal pupil diameter is 2–5 mm after light stimulus is given. Decreased pupil reactivity toward light stimulus is related to the duration and degree of increased ICP. A prospective cohort of children aged under 18 years with acute head injury and encephalopathy showed a negative correlation between increased ICP and pupil diameter (21). A prospective observational study involving 273 participants with head trauma and increased ICP showed that individuals with normal ICP (<15 mmHg) had a quicker pupil dilation response compared to those without increased ICP (>15 mmHg), without correlation with the pupil diameter (47). Several studies proved that the slow reactivity of pupils or no pupil reactivity toward light is a sign of compression at the dorsal midbrain (location of the Edinger–Westphal nucleus), defect of brain perfusion, and neurotransmitter changes related to the extent of brain lesion in the cortical area, subcortical area, and brain stem (48).

The diameters of the right and left pupils showed no significant difference before and after osmotherapy administration [3 (range 0.5–5) mm vs. 3 (range 2–5) mm]. This might be a result of the pupil diameter measurement method using a pen light. The result could be improved using quantitative methods (49).

A prospective observational study involving participants aged above 18 years receiving 20% mannitol or 3% NaCl showed that pupil reactivity toward light improved after 2 h of osmotherapy administration without affecting the pupil diameter (50). In our study, the measurement of pupil diameter and light reflex is monitored simultaneously and continuously. This research method has not been used in previous studies, which usually performed the measurement at one time point. In our study, light reflex showed a significant change with a positive correlation with ONSD at every time point of measurement. Four patients who died in this study did not respond after 48 h of osmotherapy administration, which is reflected by the ONSD value. One patient was diagnosed with encephalitis due to herpes simplex with an ONSD before and after osmotherapy of 5.14 mm for the right eye and 5.083 for the left eye and of 6.43 mm for the right eye and 6.56 mm for the left eye, respectively. Three participants were diagnosed with grade III TBM with a mean ONSD before osmotherapy of 5.66 mm for the right eye and 5.67 mm for the left eye, and mean ONSD after osmotherapy of 6.33 mm for the right eye and 6.42 mm for the left eye. The pupils of these four patients also showed no response after 48 h of osmotherapy administration. The pupil diameters of the left and right eyes in patients who were declared brain dead and died were 5 mm with negative light reflex, whereas one patient had stopped the osmotherapy 24 h after therapy due to improvements of ONSD (5.58 and 5.34 vs. 4.24 and 4.30 for right and left eyes, respectively), degree of consciousness, and light reflex. Pupil diameter and light reflex are other parameters that could be used to determine increased ICP in cases of infection and non-infection. In our study, the measurement of pupil diameter and reactivity only used a pen light, whereas other studies used automated pupillometry, which could measure the smallest or biggest pupil diameter, percentage of changes, rate of constriction, and dilation. These measures are the components of the neurological pupil index (NPI) with a normal value of ≥3 (49).

The administration of sedative agents in our study does not affect degree of consciousness, pupil diameter, and light reflex. This finding is in accordance with previous studies that found that systemic reactions of sedatives are affected by the cumulative dose of the agent in blood. The monitoring of all the patients in this study was only during 48 h of osmotherapy, which might not be enough for the agents to affect degree of consciousness, pupil diameter, and light reflex. Previous studies involving 171 adults with increased ICP showed that the concentration of benzodiazepines was not related to the degree of consciousness and pupil diameter. Apart from that, the rate of drug elimination was faster in children compared to adults (51).

Overall, our study showed a significant correlation between ONSD with degree of consciousness in patients aged 2–18 years with CNS infection after osmotherapy administration. No significant correlation between ONSD with pupil diameter in patients aged 2–18 years with CNS infection after osmotherapy.

There are several limitations to our study. First, we performed the measurement on closed eyelids, which would cause difficulties in determining the gaze direction and decrease image quality. However, performing the measurements on open eyelids would be impractical and poorly tolerated by emergency patients (52). We also used a B-scan technique, which has been associated with a blooming effect and appearance of artifacts (53). These limitations of the B-scan technique make it difficult to define the cut-off value for ONSD measurement, as various published studies found different cut-off values (36–38). A possible solution to this limitation would be to use the same B-scan device for a large number of patients and define the cut-off value for that particular device. Another possible solution is utilization of the A-scan ultrasound technique would be useful to overcome these limitations, as it does not cause a blooming effect, produces fewer artifacts, has higher sensitivity when it comes to differences less than 0.5 mm, and is more standardized compared to the B-scan technique (54). We decided to perform the measurement using the B-scan technique because it is considered easier for doctors who are not specifically trained in ophthalmologic ultrasound compared to the A-scan technique. The B-scan technique also does not require the administration of sedative agents, requiring more time for preparation. Therefore, it is more practical for measurements in emergency patients (55). Furthermore, the study was performed by only one examiner, which made it difficult to include patients who arrived at the same time in different centers. We also did not adjust the lighting quality used for measurement of pupil diameter, which could affect the results. The measurement of pupil diameter in this study was performed qualitatively using a pen light, making it difficult to accurately assess the constriction and dilation process of the pupil, which could be improved using automated pupillometry or other quantitative methods. The analysis of the effects of sedative agent administration in this study was only performed for 48 h, which makes it difficult to assess the effects of sedative agents toward ONSD, degree of consciousness, and pupil diameter.

## Data Availability

The raw data supporting the conclusions of this article will be made available by the authors, without undue reservation.
